# QoS-Oriented High Dynamic Resource Allocation in Vehicular Communication Networks

**DOI:** 10.1155/2014/718698

**Published:** 2014-01-30

**Authors:** Tarek Bejaoui

**Affiliations:** FSB, University of Carthage, 02 rue de Sfax, Bizerte 7000, Tunisia

## Abstract

Vehicular ad hoc networks (VANETs) are emerging as new research area and attracting an increasing attention from both industry and research communities. In this context, a dynamic resource allocation policy that maximizes the use of available resources and meets the quality of service (QoS) requirement of constraining applications is proposed. It is a combination of a fair packet scheduling policy and a new adaptive QoS oriented call admission control (CAC) scheme based on the vehicle density variation. This scheme decides whether the connection request is to be admitted into the system, while providing fair access and guaranteeing the desired throughput. The proposed algorithm showed good performance in testing in real world environment.

## 1. Introduction

Similar to other mobile ad hoc networks, vehicular ad hoc networks (VANETs) are self-organizing and are formed directly by a set of smart vehicles. They can interact without using fixed infrastructure or centralized administration. They have, moreover, several characteristics that distinguish them from the other networks. The mobility of vehicles in different traffic conditions, such as during traffic jams, accidents, traffic lights, and rush hours, results in the dynamic change in the network topology. In these networks, vehicles move only on predetermined roads, and they do not have the problem of resources limitation in terms of data storage and power.

In high dynamic vehicular network topology like in rural highways or during late night, the vehicles move with high speed and thus the vehicle-to-vehicle communication link remains active for short-time duration. Increasing the transmission range and then the transmission power is one of the solutions that could be considered to prolong the vehicle-to-vehicle or the vehicle to infrastructure roadside connection time. In vehicular networks the high transmission power results high interference and high network overhead in highly dense traffic (e.g., area with high penetration ratio or urban area or traffic jams). Therefore, in order to address these problems, dynamic adaptation of transmission range/power is crucial. It is required to decrease the transmission power for high vehicle density or high penetration ratio and increase the transmission power for less vehicle density or low penetration ratio. Thus, the vehicle density becomes a single metric describing the nodes' mobility in VANETs [1] that should be taken into account in any resource allocation algorithm that could be developed for these networks.

In this paper, we propose therefore a novel vehicle density-based call admission control scheme for vehicular networks that dynamically adapts vehicles' transmission powers and provides desired throughput guarantees to users performing communication within a real world-like vehicular-to-roadside IEEE 802.11p communication networks.

The paper is organized as follows. In the next section, we present an overview of the 802.11p standard and recent works devoted to CAC in VANETs, and then we highlight the impact of vehicle density on network performance. In Sections 3 and 4 we present, respectively, the new CAC function and the novel scheduling policy that we propose for VANETs. We describe our simulation platform in Section 5, in which some quantitative results are reported. Concluding remarks are presented in Section 6.

## 2. An Overview of 802.11p and CAC Implementation in VANET

In this decade, the smart vehicles become part of the Intelligent Transportation System (ITS). The MAC and physical layers of this system are supported by IEEE 802.11p Wireless Access in Vehicular Environments (WAVE) standard [2]. In this section we will briefly outline the design and concept of the WAVE standard [3] relevant to our work. The physical layer can rely on seven channels of 10 MHz bandwidth each. The spectrum of WAVE is allocated in the upper 5 GHz range.

The MAC layer in WAVE is equivalent to the IEEE 802.11e Enhanced Distributed Channel Access (EDCA) quality of service (QoS) extension. The IEEE 802.11e standard is often used for VANET prototyping implementations to support quality of service and the EDCA is the preferred channel access of this standard. It is used for service differentiation and has four access categories (AC) that support priority-based service [4]. Within the MAC layer a packet queue exists for each AC.

There is a set of EDCA parameters associated with each AC. Those parameters include arbitration interframe space (AIFS[AC]), contention window (CW) with its minimum and maximum values CWmin[AC] and CWmax[AC], and the transmission opportunity (TXOP). A backoff mechanism is defined for each AC*i* (*i* = 0,1, 2,3).

Each AC from every station starts independently a backoff timer after detecting that the channel is idle for an AIFS[AC] interval and competes with other ACs to gain a transmission opportunity. For each AC, the backoff period is selected from a uniform distribution over [0, CW[AC]]. In 802.11p, the CW size is initially assigned “CWmin” value and becomes equal to “CW = 2∗(CW + 1) − 1” when transmission fails up to the CWmax. The smaller the AIFS[AC] and the CWmin[AC] or the larger the TXOP, the shorter the channel access delay for the corresponding priority and hence the better chance to access the media for a given traffic condition.

### 2.1. Admission Control: Related Work

The VANET MAC policies proposed in the literature often take less into consideration power constraints or time synchronization problems. However, they care about the fast topology changing and the different kinds of applications for which the transmission will be established.

EDCA is very likely to be the dominant channel access mechanism in such networks because it is a distributed MAC scheme and easy to implement. Many research works are focusing on admission control in EDCA. It was proposed to administer policy or regulate the available bandwidth resources. Basically, the existing EDCA admission control schemes can be classified into two categories: measurement-based admission control and model-based admission control.

In the measurement-based schemes, admission control decisions are made on the basis of the continuously measured work conditions such as throughput and delay. In [5, 6], for example, the Distributed Admission Control policy (DAC) was proposed and for which the transmission budget for each AC is computed by subtracting the occupied time from the transmission limit of this AC.

The DAC policy can only protect existing flows when the traffic load is not very heavy. In addition, this scheme does not provide direct relationships between TXOP parameters and the QoS requirements from applications.

In [7] authors presented the Two-Based Protection and Guarantee mechanism, which is based on the DAC scheme. The purpose of the first level protection is to protect each existing voice or video flow from new and other existing QoS flows, while the purpose of the second level protection is to protect the existing QoS flows from best-effort traffic. However, this scheme has the problems of performance oscillation and lack of direct QoS relationships with applications.

The authors in [8] propose the Virtual MAC and Virtual Source Algorithms that virtually run the applications and the MAC processes in order to measure the achievable service qualities. The Threshold-Based Admission Control protocol presented in [9] takes into account the traffic condition on the wireless link. Depending on how the traffic condition is computed and measured, it can be implemented while using the relative occupied bandwidth or using the average collision ratio. In the HARMONICA scheme [10] the link-layer quality indicator (LQI) parameters are periodically sampled. They include drop rate, link layer end-to-end, and throughput for each traffic class. Two adaptation algorithms over different time scales are employed to select the channel access parameters, which can best match the QoS requirements of each traffic class and the current channel contention level. Whenever a new real-time application requires admission, HARMONICA will select a traffic class *i* that best matches its QoS requirement and then execute an admission control process. The decision of admission control is then based on the throughput requirement of the flow and the monitored LQI parameters. This is done while checking whether it is possible to squeeze some bandwidth out of the current throughput for the best effort class on the condition of guaranteeing a minimal bandwidth for this class.

On the other hand, many model-based schemes were proposed for EDCA. They define some performance metrics to evaluate the status of the network. One of such protocols is the Markov Chain Model-Based Admission control [11] in which the admission control is performed on the basis of the predicted achievable throughput for each flow. Authors in [12] propose an algorithm for providing throughput guarantee services in 802.11e EDCF wireless LANs and a Contention Window Based Admission control. Its key idea is to adjust the CW values for different stations so that the goals of admission control can be fulfilled. The main disadvantage of this scheme is to not consider the nonsaturation conditions and virtual collision, and this is the case of the Markov Chain Model Based Admission control scheme presented before.

Very few works dealing with the optimization of the minimum contention window values CWmin were presented in the literature. In [13], for example, authors consider the case where stations can have different weights corresponding to different throughput classes. Their test-bed evaluation considers both the long-term throughput achieved by wireless nodes and the short-term fairness. When all the nodes have the same transmission rate, optimality is achieved when a station throughput is proportional to its weight factor, and the optimal minimum contention windows maximize the aggregate throughput. When stations have different transmission rates, the optimal minimum contention window for high rate stations is smaller than for low rate stations.

### 2.2. Vehicle Density Impact in VANET

Common mobility models of vehicular ad hoc networks allow station mobility to be considered independently of their density in admission control protocols development and performance evaluation. In contrast, car-following models [14] show that the average speed of vehicles is a function of the vehicle density in the area. This would motivate the use of “density” as a single metric describing the nodes' mobility in VANETs.

Nodes' density has a great impact on the performance of ad hoc networks by influencing factors such as capacity, routing efficiency, robustness, and delay. Waves of traffic jams, whether caused by constraints in the transportations network, driving fluctuations, or traffic controls, cause the network density to vary from one location to another, thus disturbing the homogenous distribution of nodes. Moreover, the abrupt and frequent change in density creates a highly dynamic topology that would cause severe degradation to the network performance (increased collisions and interference, excessive broadcasts, too many routing paths, etc.) if protocols in VANETs were not designed to handle such conditions. Controlling the communication range by adjusting the transmission power can be used to mitigate the adverse effects of high density condition. The choice of the communication range has a direct impact on the connectivity which represents a fundamental property of an ad hoc network. In a VANET, a static transmission range cannot maintain the network connectivity due to the nonhomogenous vehicles distribution and rapid change of traffic conditions. In [15], authors estimate the lower and upper bounds for the transmission range and in [16] they provide a probability for gap existence among nodes. In networks of infinite size, the transmission range is related to vehicle density rather than the line length [17]. Connectivity in infinite networks is limited to short range communications, and a large-scale ad hoc network is not feasible because it is almost surely divided into an infinite number of partitions.

In [1], authors provide a relationship that allows to estimate the local density and distinguish between two phases of traffic, free-flow and congested traffic. The density estimate is used to develop an algorithm that sets a vehicle transmission range dynamically according to local traffic conditions.

On the basis of some policies presented above, we have developed a novel resource allocation scheme in the context of highly dynamic vehicular communication networks architecture. Its features are presented in the following.

## 3. Throughput Guarantee and Vehicle Density-Based CAC Algorithm: Description and Architecture

The standards 802.11e EDCA and the 802.11p that could be considered for communications in vehicular networks define a number of parameters that can be used to achieve service differentiation. However, they do not define how these parameters should depend on the network conditions like the load and the traffic characteristics in order to efficiently utilize the shared wireless channel. Moreover, common mobility models of vehicular ad hoc networks allow station mobility to be considered independently of their density in admission control protocols development and performance evaluation. However, nodes' density could have a great impact on the performance of ad hoc networks by influencing factors such as capacity, routing efficiency, robustness, and delay. This provides then the motivation to use “density” as a single metric to describe the nodes' mobility in VANETs.

In this paper, we propose then a novel CAC algorithm that provides the desired throughput guarantees on the basis of the vehicle density and the nodes' transmission range in 802.11p vehicular ad hoc networks. We consider vehicle-to-roadside (V2R) communications which are essential to properly manage traffic situations [18]. This scheme uses a cross layer approach as it adapts the transmission power (in PHY layer) and optimizes the contention window size (in MAC-layer) on the basis of the vehicle density estimation, to enhance the performance of vehicular communications.

The throughput guarantee investigation will be based on the algorithm proposed by Banchs et al. presented in [12] and that was originally performed for EDCF. It was proven to be efficient and we will adapt it in the context of 802.11e EDCA. The novelty is to take into consideration the vehicle density, as well as the transmission range of each vehicle operating within the coverage area of a road infrastructure access point (AP).

Let *r*_*i*_ be the throughput experienced by station *i*. According to Banchs et al. in [12], *r*_*i*_ is represented by the following expression:

(1) $$\begin{array}{c} r_{i}=\frac{w_{i}}{\sum_{i}w_{i}}\times \frac{l}{T_{s}-T_{c}+\left(P_{e}\cdot \left(T_{e}-T_{c}\right)+T_{c}\right)/P_{s}}, \end{array}$$

where *l* is the average payload length, *T*_*s*_ is the average duration of a successful transmission, *T*_*c*_ is the average duration of a collision, *T*_*e*_ is the duration of an idle time slot, *P*_*s*_ is the probability of a successful packet transmission, and *P*_*e*_ is the probability of an empty time slot. Consider the following:

(2) $$\begin{array}{c} w_{i}\;\text{was}\;\text{defined}\;\text{as}:\;w_{i}=\frac{\tau_{i}}{\tau_{1}}, \end{array}$$

where “*τ*_*i*_” is the probability that station *i* transmits in a generic time slot and “*τ*_1_” refers to the “*reference station*” from which the AP receives the highest signal power. And since *l*, *T*_*s*_, and *T*_*c*_ are constants, the maximum throughput experienced by all *r*_*i*_ is already defined in [12] as the following:

(3) $$\begin{array}{c} \hat{r}=\frac{P_{s}}{P_{e}\cdot \left(T_{e}-T_{c}\right)+T_{c}}=\frac{\sum_{i}w_{i}\tau_{i}\prod_{j\neq i}\left(1-w_{j}\tau_{1}\right)}{\prod_{i}\left(1-w_{i}\tau_{1}\right)\left(T_{e}-T_{c}\right)+T_{c}}. \end{array}$$

The experienced throughputs given by (1) define the set of contention window {*CW*_1_,…, *CW*_*n*_} that meets the throughput requirement set {*R*_1_,…, *R*_*n*_} for n stations and guarantee the throughput provision to them; that is,

(4) $$\begin{array}{c} \;\forall i,\in \left\{1,\ldots ,n\right\},\;r_{i}\geq R_{i}, \end{array}$$

where *r*_*i*_ represents the throughput actually experienced by station *i* and *R*_*i*_ is its throughput requirement.

**Figure 1:**
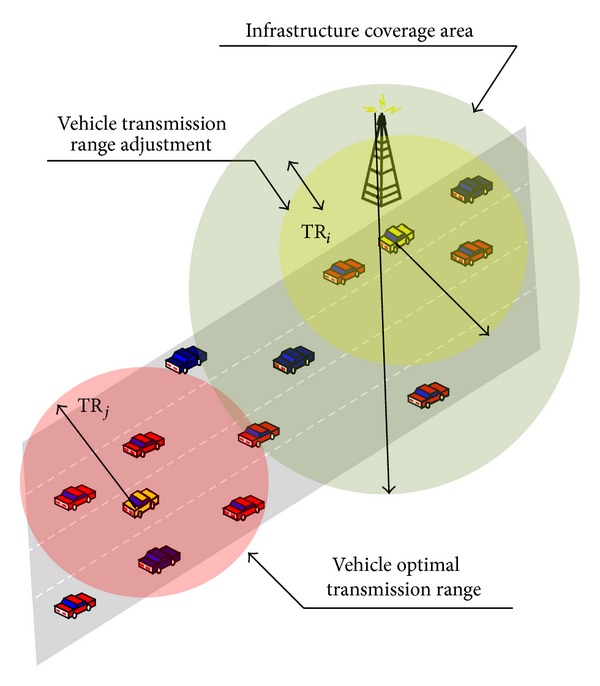
Transmission range/power adaptation based on local density.

In this paper, instead of defining one reference node for all vehicles connected to the infrastructure Access Point, on the basis of its highest throughput and highest delivered signal level, we refer to each vehicle “*i*” competing for resources within this AP coverage area (Figure 1), a reference node “ref_*i*_” [19].

The “ref_*i*_” will be then selected from the pool of the vehicles within the transmission range of the current node *i*. This would be more adequate and fairer.

The transmission range of each station operating in the network should be then computed. This is done in order to define the set of vehicles operating within its coverage area and from which the reference node will be chosen. The reference node “ref_*i*_” is always the node that uses the highest throughput and delivers the highest signal level as well.

Therefore, we consider the relationship that allows a vehicle to estimate the local density. This relationship, defined in [1], is used to set the vehicles' transmission range (TR_*i*_) dynamically and then the transmission power as depicted in Figure 1. It is a function of the vehicle local density and given by

(5) $$\begin{array}{c} \text{T}{\text{R}}_{i}=\min\left(L\times \left(1-K\right),\sqrt{\left(L\times \frac{\ln\left(L\right)}{K}+a\times L\right)}\right), \end{array}$$

where “*L*” is the length of the road segment, “*a*” is a traffic constant obtained from traffic flow theory [1, 20], and *K* is the estimated vehicle density given by

(6) $$\begin{array}{c} K={\left[\frac{u_{f}K_{j}}{\lambda^{\prime }}{\left(1-F_{s}\right)}^{n+1}+1\right]}^{-1}, \end{array}$$

where *n*  is a parameter that indicates the quality of service in the transportation network. Both *n* and *λ*′ reflect the traffic service level of the road and can be determined statistically [1].

*F*_*S*_ is the average fraction of vehicles stopped in traffic during a time window size *T*. The choice of this parameter depends highly on the rate of change in traffic conditions.

(1−*F*_*S*_)^*n*+1^ represents then the normalized average traffic speed and 1/*K*_*j*_ is the average distance from front-bumper to front-bumper between vehicles.

*u*_*f*_ is the average speed of vehicles including the stopped ones.

The vehicle density surrounding a node *i* will be the main parameter that will define its transmission range. And then its own transmission power will be calculated and dynamically adjusted accordingly.

Note that, in free-flow traffic where there are no interactions between vehicles, equation (5) cannot provide a right estimate of the vehicles' transmission range.

The throughputs “*r*_*i*_” that all communicating vehicles will receive will be optimized as well as the number of nodes to be admitted into the system. *τ*_1_ will be then replaced by *τ*_ref_*i*__ in (2) and (3).

In this paper we consider that no exponential backoff is considered. According to Bianchi in [21], “*τ*_*i*_” is given by

(7) $$\begin{array}{c} \tau_{i}=\frac{2}{{\text{CW}}_{i}+1}, \end{array}$$

where CW_*i*_ is the minimum contention window of the station *i*.

Since  *τ*_ref_*i*__ ≪ 1, the throughput experienced by a station *i* could be approximated as the following:

(8) $$\begin{array}{c} {\hat{r}}_{i}^{\text{opt}}\approx \frac{a\tau_{\text{re}{\text{f}}_{i}}-b\tau_{\text{re}{\text{f}}_{i}}^{2}}{c\tau_{\text{re}{\text{f}}_{i}}+T_{e}}, \end{array}$$

where

(9) $$\begin{array}{c} a=\sum_{i}w_{i},\;b=\sum_{i}\sum_{j\neq i}w_{i}w_{j}‍,\;c=\sum_{i}^{}w_{i}\cdot \left(T_{c}-T_{e}\right), \end{array}$$

with

(10) $$\begin{array}{c} w_{i}=\frac{\tau_{i}}{\tau_{\text{re}{\text{f}}_{i}}}. \end{array}$$

Let  *τ*_ref_i__* be the optimal value of  *τ*_ref_*i*__ that maximizes ${\hat{r}}_{i}^{\text{opt}}$. Thus, for$\;{d{\hat{r}}_{i}^{\text{opt}}/d\tau_{\text{re}{\text{f}}_{i}}|}_{\tau_{\text{re}{\text{f}}_{i}}=\tau_{\text{re}{\text{f}}_{i}}^{*}}=0$ we obtain *bc* · (*τ*_ref_i__*)^2^ + 2*bT*_*e*_ · *τ*_ref_i__* − *aT*_*e*_ = 0, and then

(11) $$\begin{array}{c} \tau_{\text{re}{\text{f}}_{i}}^{*}=\frac{\sqrt{{\left(bT_{e}\right)}^{2}+abcT_{e}-bT_{e}}-bT_{e}}{bc}. \end{array}$$

The optimal contention window set that we propose and that maximizes simultaneously all throughputs *r*_*i*_ experienced by each vehicle within the transmission range of the AP will be defined then as

(12) $$\begin{array}{c} \text{C}{\text{W}}_{i}^{\text{opt}}=\frac{2}{\tau_{i}^{*}}-1=\frac{2}{w_{i}\tau_{\text{re}{\text{f}}_{i}}^{*}}-1=\frac{2}{\left(\tau_{i}/\tau_{\text{ref}}\right)\cdot \tau_{\text{re}{\text{f}}_{i}}^{*}}-1. \end{array}$$

When a new station (*n* + 1) that requires a throughput *R*_*n*+1_ would like to join the road infrastructure, the AP first computes a new contention window set {CW_1_^opt^,…, CW_*n*_^opt^, CW_*n*+1_^opt^} using (12) and taking into account the new incoming vehicle to compute the reference node for each station. Then, it uses (8) to calculate the new throughputs that the (*n* + 1) stations would receive with this new contention window set.

If the resulting throughputs meet the requirements, that is,

(13) $$\begin{array}{c} \forall i,\in \left\{1,\ldots ,n+1\right\},\;{\hat{r}}_{i}^{\text{opt}}\geq R_{i}, \end{array}$$

then the station (*n* + 1) is accepted within the network and the new contention window set is distributed to all stations. Otherwise, the station (*n* + 1) is rejected.

Finally, it is to remind that the function of the vehicle density estimation that we have took into account is not usable in the free-flow traffic. So, the CAC scheme that we have proposed in this paper and the adaptation of the contention window are valid only in the case of dense traffic.

## 4. Priority Level Computing Function

In this section we propose a set of rules to prioritize traffic under certain conditions in the context of VANET. They are governed by an appropriately designed utility objective priority function that we have developed. This function, for which we give the acronym “PLCF” for priority level computing function, is used to make the resource allocation decision fairer. Requests' packets will be queued according to their access categories defined in 802.11p and, after, they will be served according to the EDCA scheduler after being adjusted with PLCF. Because of their importance, we choose the following network parameters for PLCF.

- Security (*S*): when the information being exchanged is confidential, packets with high encryption will be served firstly.
- Link quality (LQ): packets sent with the strongest signal strength have the highest priorities.
- Roadside conditions (*D*): available bandwidth is used to indicate the access point conditions and is a major factor especially for services belonging to access categories AC[2] and AC[3] as voice and video traffic.
- Precedence class (*P*): packets of users that have negotiated with the service provider a premium service will be served in first followed by those of medium and low priorities, when the system is close to congestion.
- Waiting time (WT): as real time services are very sensitive to access delay, packet losses are due to excessive waiting times and the higher priority is therefore given to packets having waited for the longest average time.

**Figure 2:**
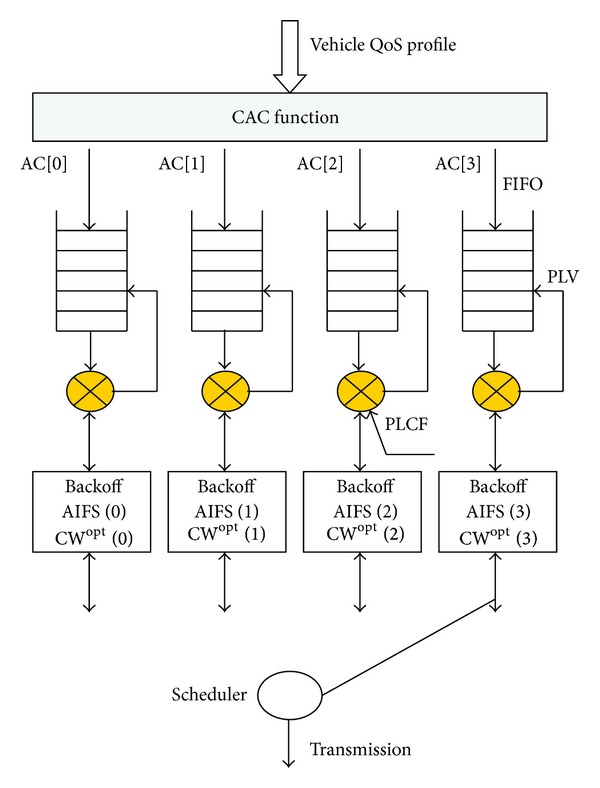
Structure of the fair packet scheduler.

PLCF is evaluated for all packets queued in buffers reserved to each vehicle access category (cf.  Figure 2). Packets with the highest calculated value for PLCF will be the first to be scheduled.

The priority level value (PLV), which provides a measure of the packet priority, is measured via the function

(14) $$\begin{array}{c} \text{PLV}=\text{PLCF}\left(S_{i},D_{i},P_{i},\text{L}{\text{Q}}_{i},\text{W}{\text{T}}_{i}\right). \end{array}$$

In order to allow for different circumstances, there is an apparent necessity to weight each factor relative to the magnitude it endows upon the priority computing function. Therefore a different weight is introduced as follows:

(15) $$\begin{array}{c} \text{PLV}=\text{PLCF}\left(w_{s}S_{i},w_{d}D_{i},w_{p}P_{i},w_{\text{lq}}\text{L}{\text{Q}}_{i},w_{\text{wt}}\text{W}{\text{T}}_{i}\right), \end{array}$$

where *w*_*s*_, *w*_*d*_, *w*_*p*_, *w*_lq_, and *w*_wt_ are weights for each of the network parameters. The values of these weights are fractions; that is, they range from 0 to 1. Furthermore all five weights add up to 1.0. Each weight is proportional to the significance of a parameter to the priority level computing. The larger the weight of a specific parameter, the more important that parameter is to the user and vice versa. These weights could be estimated and monitored by the access point of the roadside infrastructure according to the vehicle density in its coverage area; the weights that refer to the precedence class and the packets waiting time parameters will have, for example, the highest values when the system is close to congestion.

Even though we could add the different parameters in the priority level computing function to obtain packets priority level values, each vehicular network parameter has a different unit which leads to the necessity of normalization. The final normalized equation for *n* packets is

(16) PLV=wsSimax⁡⁡(S1,…,Sn)+wdDimax⁡⁡(D1,…,Dn) +wpPimax⁡(P1,…,Pn)+wlqLQimax⁡(LQ1,…,LQn) +wwtWTimax⁡⁡(WT1,…,WTn).

For each access category (AC⁡[0], AC⁡[1], AC⁡[2], AC⁡[3]) we may have two buffers so that eight buffers serving different types of traffic could be allowed to ongoing packets of each user priority defined in 802.1D.

The backoff mechanism used in the contention-based channel access is independent of the priority level computing function that we have proposed. The PLCF is then developed to provide fairness among competing 802.11p users belonging to the same access category.

## 5. Performance Evaluation

### 5.1. Simulation Platform

We investigate the adaptation of the contention window on the basis of the vehicle density in the IEEE 802.11p vehicular networks. The investigation is performed using the same traffic conditions presented in [3], while considering both time and spatial dimensions of the traffic variation. The time variation of traffic is represented as arrival process, call duration, or packet length for various types of services. Spatial variations characterize the vehicle mobility in the coverage area of 500 m of an 802.11p access point of the road infrastructure which uses an omni-directional antenna. In the real world, an 802.11p access point is expected to cover a distance that can vary between 300 and 1000 m.

In this paper, we consider that the edges of the network system are wrapped around such that the “border effect” is suppressed, as in a real world environment. As we consider dense traffic, the vehicles' speeds vary randomly between 3 km/h and 50 Km/h. These vehicles are characterized by their positions that vary each 0.5 s in the coverage area of the access point. In addition, the power transmissions vary from one terminal to another, according to their transmission range which depends on the vehicle density in the network.

We consider that signals' strength received by vehicles to define the node reference is computed while considering the three stage propagation model [22]. It takes into account the path loss, the Rayleigh fading, and the shadowing as well.

In this work, we consider that generated vehicles move in straight highway lines (cf. Figure 1) and do not change their direction as their movement is constrained by the road. In these conditions, the estimated vehicle density given by (6) will be computed using the following parameters values: *n* = 0 and 1/*λ*′ = 2 s [1].

The average fraction “*F*_*S*_” of vehicles stopped in traffic is defined during a time window size *T* = 10 s. It results in high correlation between the actual density and its estimate.

We neglect the interference produced by each vehicle on other stations in the network and we chose a packet size of 1500 bytes, since this is a reasonable average packet size including data and security information.

We assume that all stations are greedy; that is, they always have packets to transmit. We consider two groups of stations with different throughput requirements, *R*_1_ = 100 Kbps and *R*_2_ = 200 Kbps. They are competing to access to 7 channels as defined in IEEE 802.11p standard.

In this paper, we consider that the traffic generated by each communicating vehicle consists of periodic broadcast message (PBM) as it roughly accounts for 95% of the traffic in VANET. It is not time-critical but loss-sensitive service.

Since we use a discrete time simulator, the interarrival of vehicles sending PBM is Poissonly distributed with a mean value ranging between 0 and 120 s.

The value of traffic constant used in (5) is assumed to be *a* = 0.25 as per the traffic flow theory [1], and *L* = 500 m is the road segment length that is assumed to be equal to the transmission range of the 802.11p access point.

### 5.2. Simulation Results

The simulations were performed while using the Network Simulator tool NS2, to evaluate the effectiveness of the proposed resource allocation policy.

**Figure 3:**
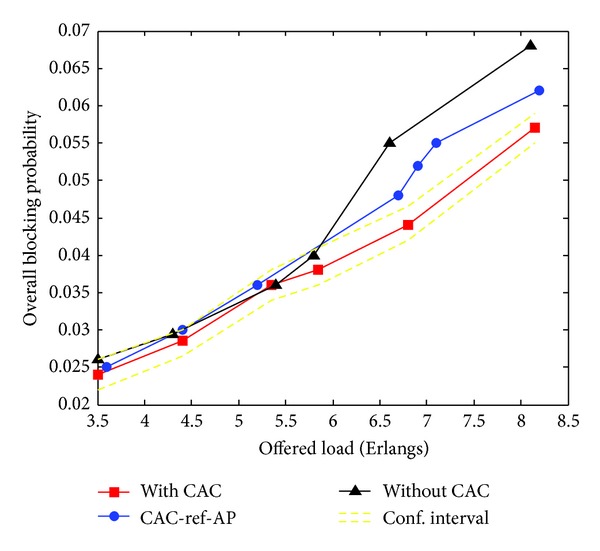
Overall blocking probability versus offered load.

Figure 3 reports the impact of the proposed CAC scheme on the overall blocking probability. At a load of 8 Erlangs, for example, the blocking probability decreases from about 6% when the reference node is chosen by the AP (CAC_reference AP) to about 5.4% when it is chosen from the pool of vehicles in the transmission range of the current station.

When no CAC scheme is applied, the overall blocking probability at 8 Erlangs is about 6.7%.

**Figure 4:**
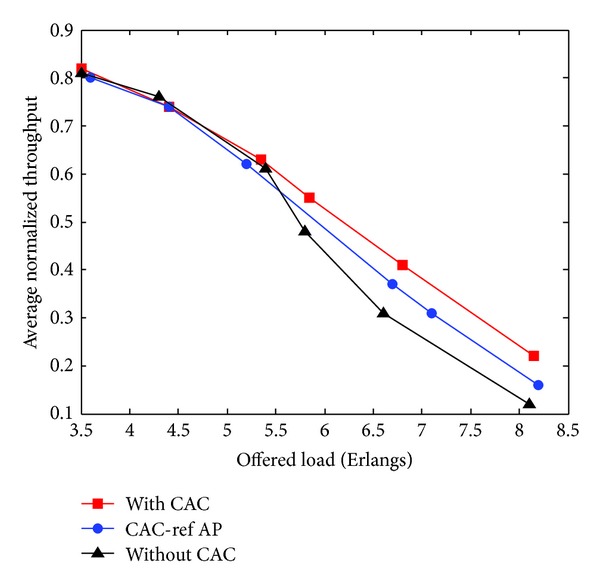
Average normalized throughput versus offered load.

**Figure 5:**
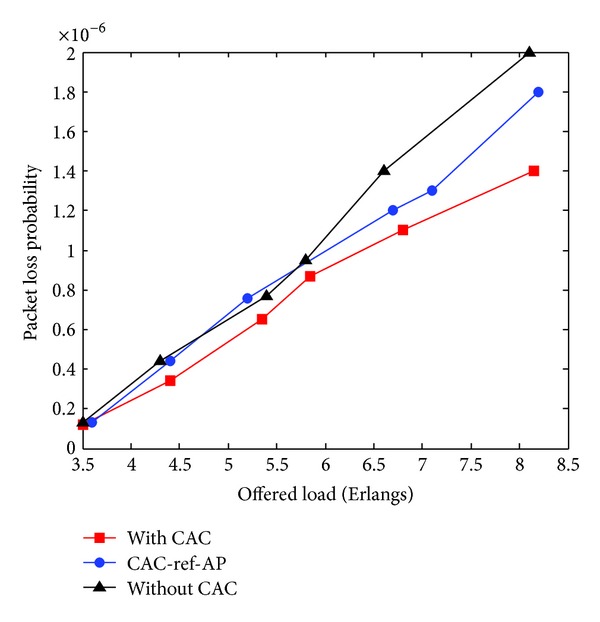
Packet loss probability versus Offered load.

Figure 4 exhibits the average normalized throughput, and since PBM is a loss-sensitive service, we report in Figure 5 the packet loss probability. These figures clearly show that the proposed CAC policy achieves a good performance and capacity gain.

We can intuitively conclude that, when the reference node is chosen by a vehicle from the pool of nodes in its transmission range rather than choosing it by the AP from the pool of all accepted nodes, CWmin will be smaller and then the channel access delay will be shorter for the corresponding service. This provides better chance to access the media for a given traffic condition.

**Figure 6:**
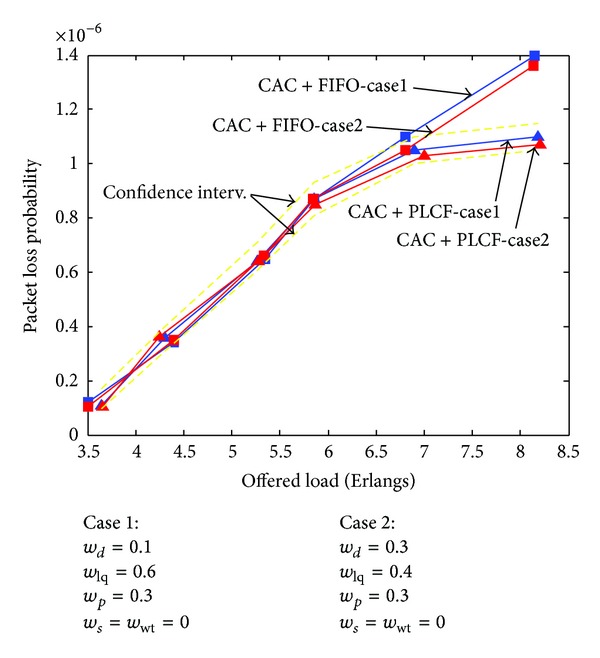
Performance gain with PLCF function.

In Figure 6 we compare the performance of the proposed CAC scheme while packets are queued according to FIFO scheduling policy or using PLCF function that adjusts the PBM packets priority level values. To show the effectiveness of PLCF, the simulations were performed by giving to all users the same weights for the security parameter, and the waiting time factor since PBM is not a time-critical service. This is as to consider *w*_*s*_ = *w*_wt_ = 0. The three remaining parameters in the PLCF function are then the link quality, the roadside conditions, and the precedence class. PBM is not bandwidth hungry; that is, bandwidth could not be the most significant parameter, and thus, users of this type of service should be given low weights. In this work, simulations were performed considering two cases, where *w*_*d*_ = 0.1 and *w*_*d*_ = 0.3. The weight *w*_lq_ relative to the signal strength is equal to 0.6 and 0.4, respectively. The weight *w*_*p*_ for the precedence class is admitted to be constant and then equal to 0.3. The results show that, in case 2, the packet loss probability is almost equal or slightly lower than in case 1 at high traffic load. This is because stations using higher throughputs are served in first and the most of their packets will not wait beyond the limit of time.

By implementing the PLCF, the system manages to decrease the packet loss probability by up to 22%. For example, for loss-sensitive services like PBM using PLCF-based scheme, it shows a significant improvement in performance in VANETs. This improvement is made clear for high traffic load (from about 7 Erlangs).

The validation of results by analytical model is very difficult since the full details of a scheduling policy and a dynamic CAC function with users' mobility and propagation environment cannot be described by formulas usable in practice.

The simulation run takes duration of about (10^6^ × simulation time unit) to achieve a confidence level of 95%.

## 6. Concluding Remarks

In this paper we presented a cross-layered admission control algorithm based on throughput guarantee and dynamic adaptation of joint transmission range and contention window according to the vehicle density and network traffic conditions. A priority function based on significant vehicular network parameters to make the scheduling decision fairer is proposed as well.

Results have shown that our scheme proposed for the IEEE 802.11p vehicular networks improves the performance of the vehicular communication and achieved capacity gains.

Future work includes the performance analysis of the proposed scheme for each type service independently while taking into consideration more constrained movement. The EDCA scheduler will be substituted by other schedulers like those based on WRR and WFQ scheduling policies. A comparison investigation could be provided for all classes of services.
